# Locating the mental foramen at the bedside with point of care ultrasound imaging

**DOI:** 10.11604/pamj.2018.29.54.10493

**Published:** 2018-01-21

**Authors:** Abdullah Ebrahim Laher, Feroza Motara1, Mike Wells

**Affiliations:** 1 Department of Emergency Medicine, University of the Witwatersrand, Faculty of Health Sciences, Johannesburg, South Africa

**Keywords:** Mental foramen, mental nerve, ultrasound, point of care, emergency department

## Abstract

**Introduction:**

Ultrasound guided neuro-anaesthesia is a developing field of interest to clinicians from various disciplines. The objective of this proof of concept study was to explore the ability, ease and rapidity of ultrasonography in locating the mental foramen.

**Methods:**

A convenience sample of 100 patients aged 18 years or older, with no known pathology to the mandibular region, that presented to a single urban ED were enrolled. All patients underwent an ultrasound examination on both sides of the face to locate the mental foramina.

**Results:**

A total of 100 patients' mental foramina were studied. Mean age was 35.7 years (SD 9.1 years), 50% were black and 25% each were asian and white. The mental foramina were ultrasonographically identified in all (100%) of the subjects in the study group. Although requiring a larger quantity of ultrasound gel, the mental foramina were also visualized in all twelve subjects with facial hair. Three out of the 100 subjects were noted to have accessory mental foramina. The overall mean time taken to locate the first mental foramen in each patient was 16.1 seconds (SD 12.9 seconds). For the first 25 subjects studied, the mean time taken was 34.7 seconds (SD 13.4 seconds), whereas for the next 75 subjects studied, the mean time taken was 9.9 seconds (SD 3.0 seconds).

**Conclusion:**

Bedside ultrasound imaging is a potentially reliable method to identify and locate the mental foramen. With practice and experience, the mental foramen can be more easily identified.

## Introduction

The mental foramen is found on the mandible, in the vicinity of the second premolar tooth. The mental nerve, which is a terminal branch of the inferior alveoli nerve, emerges from the mental foramen together with the mental vessels. The mental nerve and its branches provide sensation to the area around the angle of the mouth, the oral mucosa and gingiva as far posterior as the 1st molar as well as the skin of the lower lip and mental region [1,2]. The mental foramen and the various modalities useful in locating the mental foramen have been reviewed in detail elsewhere [3]. Individuals presenting to the Emergency Department (ED) may require anaesthesia for simple and complex procedures to the field of supply of the mental nerve. Unlike oral health practitioners who regularly perform mental nerve blocks, the ED practitioner is less likely to be familiar with the anatomical landmarks of the mental nerve. Potential complications of a mental nerve block include inadvertent intravascular administration of the local anaesthetic agent, local anaesthetic toxicity, haematoma formation, mental nerve injury and a failed nerve block [4]. Therefore, accurately determining the position of the mental foramen would allow for the safer administration of the appropriate dose of local anaesthetic at the correct place. This may potentially decrease complication rates and improve first time success rates. The use of ultrasonography for regional nerve block anaesthesia, has been shown to be safe, effective, accurate and time saving [5]. With the ever increasing scope of Emergency Medicine, Emergency Physicians now competently perform a variety of ultrasound guided diagnostic and interventional procedures in the ED [6]. These Emergency Physician led procedures positively impact on time saving, cost saving, ED overcrowding and patient satisfaction [7]. The non-invasiveness, safety, lack of radiation exposure, cost saving and portability of point of care bedside ultrasound makes it an appealing diagnostic and interventional modality in medicine [8] and more specifically in the ED. The aim of this proof of concept study is to firstly prove the ability of ultrasound to safely and accurately locate the mental foramen. We also determined the effect of experience on the ability and rapidity in locating this anatomic landmark of interest.

## Methods

**Figure 1:**
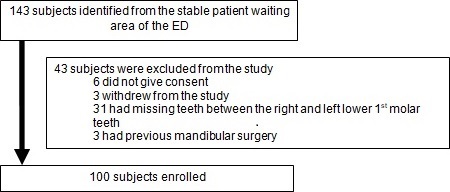
Exclusions and final sample for analysis

We conducted a prospective, cross-sectional study on patients presenting to a single center ED (Charlotte Maxeke Johannesburg Academic Hospital (CMJAH), Johannesburg, South Africa) between February 2012 and March 2013. CMJAH is a tertiary care facility in a large urban environment affiliated with the University of the Witwatersrand. A convenience sample of 100 Black and Caucasian (White and Asian) adults, older than 18 years were enrolled. These patients had passed through the hospital's triage system and did not require urgent or emergent treatment. Our primary objective was to ultrasonographically locate the mental foramina in each participant. We also assessed the rapidity in identifying the mental foramen, the effects of facial hair and the presence of accessory mental foramina. Individuals with congenital / acquired facial distortion, a history of mandibular surgery and patients who had mandibular teeth missing between the right and left lower 1st molars were excluded from the study. Details of patient recruitment and exclusion are listed in the flow diagram in Figure 1.

**Figure 2:**
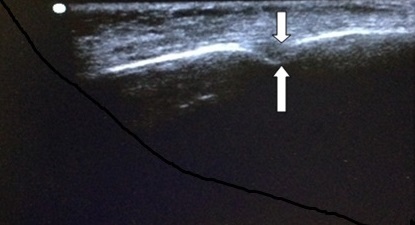
Ultrasound image of the mental foramen (between white arrows); the mental foramen is easily identified as a break in the continuity of the bone in the vicinity just below the corner of the mouth

All ultrasound examinations were conducted by a certified point of care ultrasound practitioner. The study was further fully supervised by a faculty member aligned with the division of point of care emergency ultrasound. Subject recruitment was conducted by a second investigator who had no involvement with the actual data collection procedure. Patients were randomly selected from the queue at a time convenient to the researchers. Subjects, who consented to participate in the study, were asked to lie in the supine position in an examination cubicle. All ultrasound investigations were performed with the patient lying in the supine position. We used a Toshiba diagnostic ultrasound system (model SSA-510A; Toshiba; Tokyo; Japan), a high-frequency (8-MHz) transducer (PLF-805ST; Toshiba; Tokyo; Japan) and ultrasound conductive gel (Konix^®^ ultrasound gel, Sanichem, Durban, South Africa). A second certified point of care ultrasound practitioner initiated the timer prior to the primary investigator applying the ultrasound transducer to the subjects face with the marker directed cranially. The mental foramen was identified as a break in the continuity of the bone. There are no other similar ultrasonographic structures that may be confused with the mental foramen in this region (Figure 2). Once the second investigator had confirmed the correct image, the timer was stopped and time taken to identify the mental foramen recorded. All volunteers, as far as possible, maintained their position in the queue waiting to see their clinician. Any difficulties experienced during the investigation were documented. As part of the same study we also described the relationship of the mental foramen to soft tissue landmarks [9], hard tissue landmarks [10] as well as the mandibular premolar teeth [11]. These results have been separately reported.

**Statistical analysis:** All data was captured from the data collection sheets by the primary investigator and entered into an electronic spreadsheet (Microsoft^®^ Excel^®^). Blinded double entry was performed for all patients to ensure data integrity. STATA^®^ version12 software was used to perform all statistical analyses. Each volunteer was placed in to an age category: a) 18-30 years, b) 31-40 years, c) 41-50 years, d) 51-60 years, e) 61-70 yrs. Categorical variables such as sex, race, and age category are described with the aid of a percentage frequency distribution table. The mean distance from the mental foramen to the accessory mental foramen was calculated for the relevant subjects. The time taken (in seconds) to locate the first mental foramen in each of the 100 subjects is illustrated by means of a two-way scatter plot.

**Ethics:** Patients were enrolled in the study once written informed consent was obtained. The informed consent leaflet also included consent for publication and reporting of data. Patient confidentiality was respected at all times. Data collection sheets did not include personal data. Only relevant demographic data was included. The information gathered was protected by a coded numbering system, which was stored in a password-protected computer that was only accessible to the researchers. Permission to conduct the study was obtained from the head of department (H.O.D) of the CMJAH ED and the hospital management. Clearance was obtained from the Human Research Ethics Committee of the University of the Witwatersrand (certificate no. M110920).

## Results

**Figure 3:**
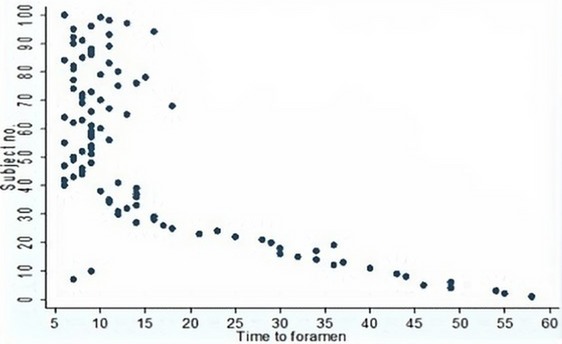
Two-way scatter plot of the time taken (seconds) to locate the first mental foramen in each of the 100 subjects

One hundred patients were enrolled in the study. This included 50 Black (27 males and 23 females) and 50 Caucasian (23 males and 27 females) subjects. The Caucasians were further subdivided into 25 Asian (13 males and 12 females) and 25 White (10 males and 15 females) subjects. The overall sex distribution was equal and comprised 50 males and 50 females. The analysis of each race group according to age is shown in Table 1. The overall mean age of the study population was 35.7 years (SD 9.1 years). Most of the subjects were aged between 18 and 30 years (40 subjects). The 31-40 year age group formed the second largest group (29 subjects). Thirty-one subjects were older than 40 years of age. The mental foramina were identified ultrasonographically in all (100%) of study participants. Three out of the 100 subjects were noted to have accessory mental foramina. They were each located 5.9 mm; 6.1 mm and 6.4 mm medial to the non-accessory mental foramen (mean 6.1 mm). All accessory mental foramina were located in the same horizontal plane as the non-accessory mental foramen. The mental foramen that was symmetrical to the opposite side was regarded as the non-accessory mental foramen. The race and gender distributions for subjects with accessory mental foramina comprised 2 black males and 1 white female. Twelve out of 100 subjects were noted to have facial hair in the region of the mental foramen. In all of these subjects the mental foramen was easily visualized with ultrasonography. However approximately double the quantity of sonar gel was utilized to achieve an acceptable image on the monitor screen. The overall mean time taken to locate the first mental foramen studied in each patient was 16.1 seconds (SD 12.9 seconds). The quickest time was noted as 6 seconds and the longest time was noted as 58 seconds. For the first 25 subjects studied, the mean time was 34.7 seconds (SD 13.4 seconds), whereas for the next 75 subjects studied, the mean time taken was 9.9 seconds (SD 3.0 seconds). This is illustrated in Figure 3.

**Table 1:** Age group frequencies for the various race groups studied

Race	Age Group					
	(18-30yrs)	(31-40 yrs)	(41-50 yrs)	(51-60 yrs)	(61-70 yrs)	Total
Black	18	20	8	3	1	50
Asian	13	6	3	3	0	25
White	9	3	5	7	1	25
Total	40	29	16	13	2	100
Mean age (yrs)	24.3	36.7	46.7	56.2	63.0	35.7

## Discussion

All mental foramina (100%) in the study group were clearly identified with ultrasound imaging. In a previous study, the mental foramen was visualized on panoramic radiographs in 94% of cases. However, less than half of these were clearly visible [12]. Other studies using panoramic and apical radiographs visualized the mental foramen in 46.8% and 84.2% of cases [13,14]. Therefore ultrasonography may be regarded as superior to panoramic and periapical films in its ability to identify the mental foramen. Also the fact that ultrasound is non-ionizing, makes it a safer option. Three percent of subjects were noted to have accessory mental foramina. This is in keeping with previous literature reports. The incidence of accessory mental foramina has been reported as between 3% and 10% [2,15]. The mean distance between the mental foramen and accessory mental foramen in our study was 6.1 mm. Naitoh et al in his study found the mean distance to be 6.3mm [16]. The importance of assessing for the presence of accessory mental foramina is to avoid infiltration of local anesthetic in the region of the empty foramen and thereby also decreasing the risk of a failed nerve block anesthesia. With the aid of larger quantities of sonar gel, the mental foramen was detected in all 12% of subjects who had facial hair present in the vicinity of the mental foramen. This suggests that the presence of facial hair should not be regarded as a contraindication or limitation to the use of ultrasound.

For the first 25 subjects studied, the mean time to detection of the mental foramen was 34.7 seconds (SD 13.4 seconds), whereas for the next 75 subjects studied, the mean time taken was 9.9 seconds (SD 3.0 seconds). Therefore, with practice and experience, the time taken to identify the mental foramen with ultrasonography was markedly shorter (Figure 3). Hence, it can be concluded that the idiom “practice makes perfect” is true. We excluded thirty four patients who mostly had missing teeth between the right and left lower 1st molars or who had a previous history of mandibular surgery. It is possible that the mental foramen may not have been as easily identified with ultrasound in these patients. Previous studies have reported the effects of ageing, tooth wear and bone resorption as a result of tooth loss on positioning of the mental foramen [1,17]. A study showed that the mental foramen was situated approximately 3.8 mm lower in edentulous jaws [18]. Theoretically the use of ultrasonography for mental nerve blocks would decrease local anesthetic requirements, increase first time success rates and decrease complication rates. Surprisingly, Tarsia and colleagues concluded that local infiltration of anaesthesia for facial lacerations was less painful and resulted in more effective anaesthesia than percutaneous regional nerve block anaesthesia [19]. However, they used a blind regional nerve block technique, which could possibly have resulted in infiltration of local anaesthesia at the incorrect position. Perhaps the use of ultrasound guided nerve block anaesthesia may have resulted in improved outcomes. Although the mental nerve is only a single anatomical entity that is relevant to the emergency department clinician, it is hoped that this proof of concept, bedside ultrasound based study stimulates more interest and research in the field of ultrasound guided neuro-anaesthesia. Future larger scale well designed studies are required to assess and compare complication rates, volume of local anesthetic agent requirements and success rates between ultrasound guided and blind nerve blocks techniques involving not just the mental nerve, but other nerves of importance to the field of regional nerve block anaesthesia.

## Conclusion

Bedside ultrasound imaging is a potentially reliable method to identify and locate the mental foramen. With practice and experience, the mental foramen can be more easily identified.

### What is known about this topic

- The mental foramen is an important landmark to clinicians that include oral clinicians, emergency medicine clinicians, family practitioners and plastic surgeons;
- The literature regarding the role of ultrasound in locating the mental foramen and other neuro-anatomy of interest has not been well reported.

### What this study adds

- Ultrasound imaging is feasible in accurately locating the mental foramen;
- With practice and experience, the mental foramen can be more easily identified.

## Competing interests

The authors declare no competing interests.
